# Homogeneous material based acoustic concentrators and rotators with linear coordinate transformation

**DOI:** 10.1038/s41598-021-91146-x

**Published:** 2021-06-01

**Authors:** Huaping Wang, Lei Zhang, Shahnawaz Shah, Rongrong Zhu, Bin Zheng

**Affiliations:** 1 Key Laboratory of Ocean Observation-Imaging Testbed of Zhejiang Province, Institute of Marin Electronics Engineering, Ocean College Zhejiang University, Hangzhou, 310058 People’s Republic of China; 2 College of Information Science and Electronic Engineering Zhejiang University, Hangzhou, 310058 People’s Republic of China; 3 School of Information and Electrical Engineering, Zhejiang University City College, Hangzhou, 310015 People’s Republic of China

**Keywords:** Applied physics, Acoustics

## Abstract

With the ability to focus and rotate the acoustic field in a given region while keeping the outside region unchanged, the acoustic concentrator and rotator has been developed for the versatile manipulations of acoustic wave. In this letter, we report the design of acoustic concentrator and rotator facilitated by linear coordinate transformation. Compared with the previous ones that have inhomogeneous parameter distributions, the designed devices are composed of several parts with homogeneous parameters, which can be achieved with the help of few homogeneous layered structures. Simulations are also performed to verify the functions of the designed device. The proposed acoustic concentrators and rotators would be useful in numerous applications such as acoustic sensing and communication.

## Introduction

With the ability to freely manipulate the propagation of electromagnetic waves in a well-defined manner, the theory of Transformation Optics (TO)^1^ has facilitated the design of novel electromagnetic devices. TO is a strategy that can control the propagation of the electromagnetic wave due to the invariance of Maxwell’s equations under the coordinate transformation. It has motivated a series of researches^2^, such as cloaks^3–6^, illusion devices^7,8^, lenses^9,10^ and antennas^11^. Besides, the concentrators^12^ and rotators^13^, which can increase and rotate the EM energy in the inner region, have also attracted remarkable attention.

Since the emergence of such concepts, various works were carried out and extended to acoustic fields^14–17^. By introducing the invariance of Maxwell’s equations into the elastic wave field, Milton proposed an elastic wave cloaking^15^. Then, Cummer and Schurig designed the first acoustic cloak by analogy the fluid acoustic wave equations and Maxwell equations under TE polarization wave^16^. The earliest and typical application of transformation acoustics is acoustic invisibility cloak^15–17^. It’s no doubt that the novel concentrator^18^ and rotator^19^ first appeared in the field of TO. It wasn't long before it was introduced into acoustics. In 2011, Yang designed an acoustic concentrator^20^ based on coordinate transformation. In 2014, Jiang designed the inhomogeneous acoustic rotator^21^. And by applying acrylonitrile butadiene styrene plastic structure, they constructed it physically. Then, more researches about acoustic concentrators^22,23^ and rotators^21,24,25^ emerged, that can magnify the intensity or rotate the propagation direction of the acoustic wave in the internal region and keep external field unchanged. However, the physical realization of the acoustic concentrators and rotators suffers from complicated parameters, especially inhomogeneity, making it difficult for experimental realization.

In this paper, we proposed acoustic concentrator and rotator based on linear coordinate transformation. By dividing the devices into polygon subregions, the transformation would be linear. Conventional cases required the parameters to be discretized and approximated with different materials. The linear coordinate transformation provides a feasible way for the design of such devices with homogeneous parameters directly. Using effective medium theory, the design can be realized by multilayered structures with only a few kinds of materials. The performances of the designed devices are validated with numerical simulations, which show good agreement with the theoretical prediction. This method can be extended to other acoustic devices, which may find potential applications in various areas e.g. underwater communication and medical ultrasound therapy.

## Methods

**Figure 1:**
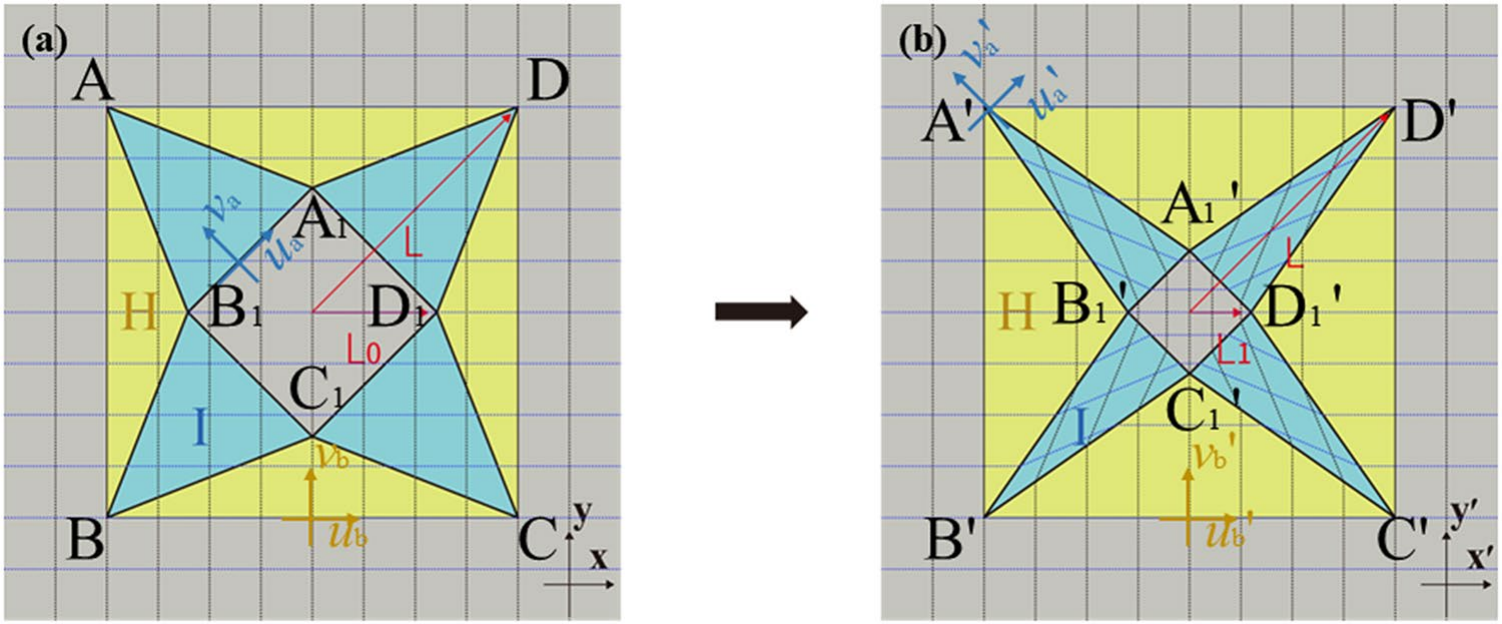
Schematic diagram of coordinate transformation for the case of square acoustic concentrator. A big square ${\text{ABCD}}$ wraps a small square ${\text{A}}_{1} {\text{B}}_{1} {\text{C}}_{1} {\text{D}}_{1}$. The annular area around the small square is split into segment I and segment II. The external diameter of big square is $L$. (**a**) Virtual space. The size of internal square is $L_{0}$. (**b**) Physical space. The size of internal square is $L_{1}$. With the shrinkage of inner small square, segment I and segment II should be transformed accordingly.

To start with, we apply this linear coordinate transformation to the design of concentrator, as shown in Fig. 1. Figure 1a shows the virtual space filled with background media, where the black line represents the outline of the different subdomains. Figure 1b shows the physical space after the coordinate transformation. The divided subdomains segments I, II and central square region are compressed or stretched individually. Among them, the transformation of the central region is the core and also the basis of the whole transformation. From the comparison of Fig. 1a,b, the ratio of compression along the x and y coordinate directions is the same in central region. For segment I and segment II, taking $\Delta {\text{AA}}_{1} {\text{B}}_{1}$ and $\Delta {\text{AA}}_{1} {\text{D}}$ as representatives, the reduction of central square results in the increase of the distance from A to ${\text{A}}_{1} {\text{B}}_{1}$ and the decrease of ${\text{A}}_{1} {\text{B}}_{1}$ in $\Delta {\text{AA}}_{1} {\text{B}}_{1}$. The compression of the central square in the y-direction of the global coordinate system results in the increase of the distance from ${\text{A}}_{1}$ to AD of $\Delta {\text{AA}}_{1} {\text{D}}$. The local coordinate systems of segment I and segment II are shown in Fig. 1. The specific transformation equations are shown as follows,

1 $$ \begin{aligned} & {\text{For}}\;{\text{region}}\;{\text{I}}:\quad \quad u_{a}^{{\prime}} = \frac{{u_{a} }}{{k_{1} }},{ }\quad v_{a}^{{\prime}} = \frac{{v_{a} }}{{k_{2} }},{ }\quad w_{a}^{{\prime}} = w \\ & {\text{For}}\;{\text{region}}\;{\text{II}}:\quad \quad u_{b}^{{\prime}} = u_{b} ,\quad { }v_{b}^{{\prime}} = \frac{{v_{a} }}{{k_{3} }},\quad { }w_{b}^{{\prime}} = w \\ & {\text{For}}\;{\text{central}}\;{\text{square}}:\quad \quad u_{c}^{{\prime}} = \frac{{u_{c} }}{{k_{1} }},\quad { }v_{c}^{{\prime}} = \frac{{v_{c} }}{{k_{1} }},\quad { }w_{c}^{{\prime}} = w \\ \end{aligned} $$

where $k_{1}$, $k_{2}$ and $k_{3}$ are compression ratios of corresponding regions: $k_{1} = \frac{{L_{0} }}{{L_{1} }},{ }k_{2} = \frac{{L - L_{0} \sin \left( \theta \right)}}{{L - L_{1} \sin \left( \theta \right)}},$ ${ }k_{3} = \frac{{\left( {L\sin \left( \theta \right) - L_{0} } \right)}}{{\left( {L{\text{sin}}\left( \theta \right) - L_{1} } \right)}}$. $\theta$ denotes the half of quadrilateral center angle; $L_{0}$ and $L_{1}$ are half the diagonal of the inner square before and after transformation respectively, while *L* is half the diagonal of outer square.

According to the transformation functions of Eq. (1), the required mass density tensor $\uprho$ and bulk modulus B of each segment can be calculated as,

2 $$ \begin{aligned} & {\text{For}}\;{\text{ region}}\;{\text{ I:}}\quad \quad \rho_{u}^{{{{I^{\prime}}}}} = \rho_{0} \frac{{k_{1} }}{{k_{2} }}, \quad \rho_{v}^{{{{I^{\prime}}}}} = \rho_{0} \frac{{k_{2} }}{{k_{1} }}, \quad {\text{B}}^{{{{I^{\prime}}}}} = {\text{B}}_{0} \frac{1}{{k_{1} k_{2} }} \\ & {\text{For}}\;{\text{ region }}\;{\text{II:}}\quad \quad \rho_{u}^{{{\text{II}}^{{\prime}} }} = \rho_{0} \frac{1}{{k_{3} }}, \quad \rho_{v}^{{{\text{II}}^{{\prime}} }} = \rho_{0} k_{3} , \quad {\text{B}}^{{{\text{II}}^{{\prime}} }} = {\text{B}}_{0} \frac{1}{{k_{3} }} \\ & {\text{For }}\;{\text{central}}\;{\text{ square:}}\quad \quad \rho_{u}^{{{{c^{\prime}}}}} = \rho_{0} , \quad \rho_{v}^{{{{c^{\prime}}}}} = \rho_{0} ,\quad {\text{B}}^{{{{c^{\prime}}}}} = {\text{B}}_{0} \frac{1}{{k_{1}^{2} }} \\ \end{aligned} $$

**Figure 2:**
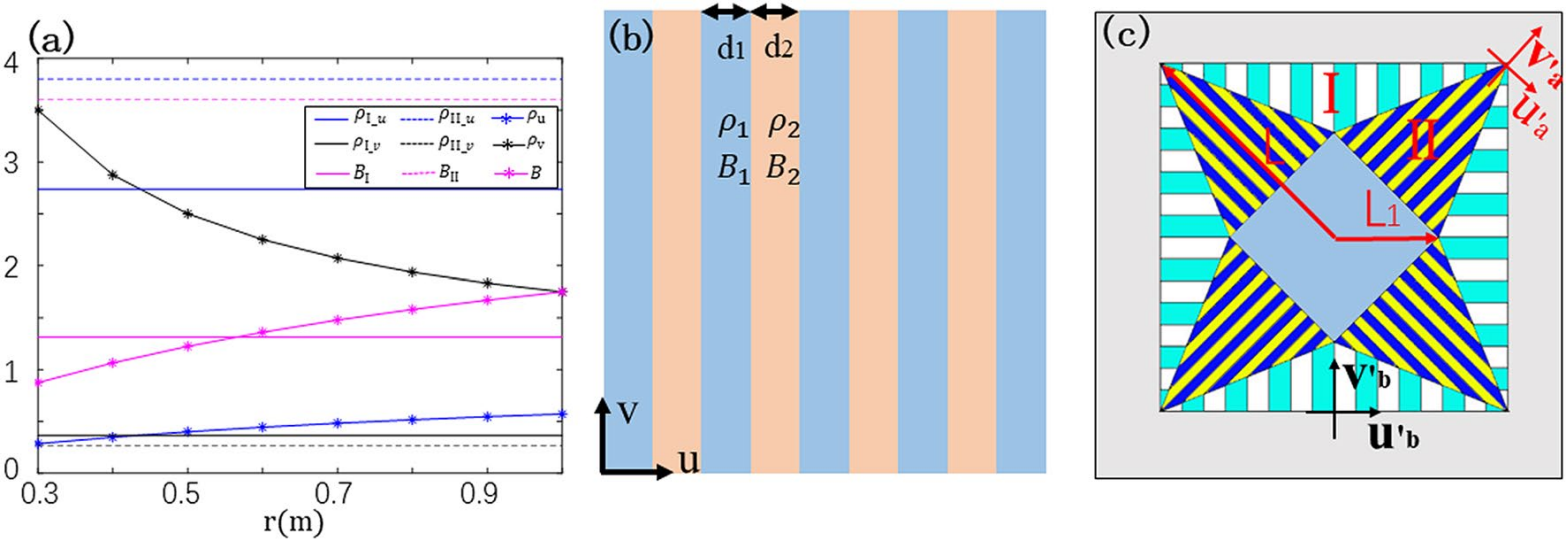
(**a**) Distribution of density and bulk modulus between our own design and Ref. 23. Normalized density components in u and v directions and bulk modulus are drawn in blue, black and carmine lines respectively. The dash dot lines represent the parameters of Ref.^23^ while straight lines and dotted lines are normalized densities and bulk modulus of region I and region II of our design. The horizontal ordinate represents the distance to the center of the device, and vertical ordinate is the normalized ratio; (**b**) geometric sketch of layered structure. Two structures with different density and bulk modulus are arranged alternately; (**c**) the layered structure schematic of the acoustic concentrator. $L_{1}$ and *L* are the boundary dimension of the inner and outer quadrilateral: $L_{1} = 0.3\;{\text{m}}, L = 1.0\;{\text{m}}$.

Equation (2) suggests that parameters are independent of the spatial coordinates, since $k_{1} ,{ }k_{2} { },{ }k_{3}$ is only related with $L,L_{0} , L_{1} { }$ and $\theta$. Therefore, the parameters possess homogeneous property. One can observe that in our device both the densities and bulk modulus for each segment are constant values, as shown in Fig. 2a straight lines and dotted lines. This characteristic is the main difference comparing to the previous Wang’s design^23^ which used normal transformation method. The density tensor and bulk modulus for the Ref. 23 are described by dash dot lines, seeing in Fig. 2a. They are inhomogeneous and require discretization since parameters are coordinate dependent. For comparison, our lines are all straight, that is, parameters remain constant. Therefore, the realization of our designed concentrator has more simplified parameters that can be achieved with few materials.

Although we realized the homogeneous parameters, densities of each segments are still anisotropic. It’s difficult to achieve physically. But layered structures consisted of a series of periodically arranged units with sizes much smaller than wavelength, as shown in Fig. 2b, can be utilized to satisfy the requirement of anisotropic parameters. According to effective medium theory^26^ for layered structure, the effective density tensors and bulk modulus for a whole structure are:

3 $$ \begin{aligned} & \left( {1 + {\upeta }} \right)\rho_{u} = \rho_{1} + \eta \rho_{2} \\ & \frac{1 + \eta }{{\rho_{v} }} = \frac{1}{{\rho_{1} }} + \frac{\eta }{{\rho_{2} }} \\ & \frac{1 + \eta }{B} = \frac{1}{{B_{1} }} + \frac{\eta }{{B_{2} }} \\ \end{aligned} $$

where ${\upeta }$ is the thickness ratio of two materials. Taking ${\upeta } = d_{1} /d_{2}$ and setting the bulk modulus of the two materials to be the same, by solving Eq. (3), we can obtain:

4 $$ \begin{aligned} & \rho_{1} = \rho_{u} + \sqrt {\rho_{u}^{2} - \rho_{u} \rho_{v} } \\ & \rho_{1} = \rho_{u} - \sqrt {\rho_{u}^{2} - \rho_{u} \rho_{v} } \\ & B_{1} = B_{2} = B \\ \end{aligned} $$

Thus, for the Segment I and Segment II, we can use layered structures to construct the concentrator, as shown in Fig. 2c. Different small strips are segmented to represent two different materials. One can see that fewer kinds of materials would suffice, making the concentrators much easier for construction. For details, the layered structures are stacked along u-directions in their own coordinate system in segment I and II, respectively, the parameters of which are calculated from Eq. (4). It should be noted that the thickness of the monolayer must be much smaller than the wavelength, according to the requirement of effective medium theory to achieve the anisotropic property. The central region is concentrating area with isotropic parameters, which is not replaced by layered structure.

Although it shows only a 2D case of the concentrator, this method is also suitable for 3D case (see Supplementary Materials). Additionally, there are two kinds of space transformation in the new transformation acoustic devices: one is the space stretching or compression, and the other is the space transformation without compression or compression. The concentrator mentioned above belongs to the first transformation mode, while the other mode can be used to realize different space transformation effect, such as rotation.

**Figure 3:**
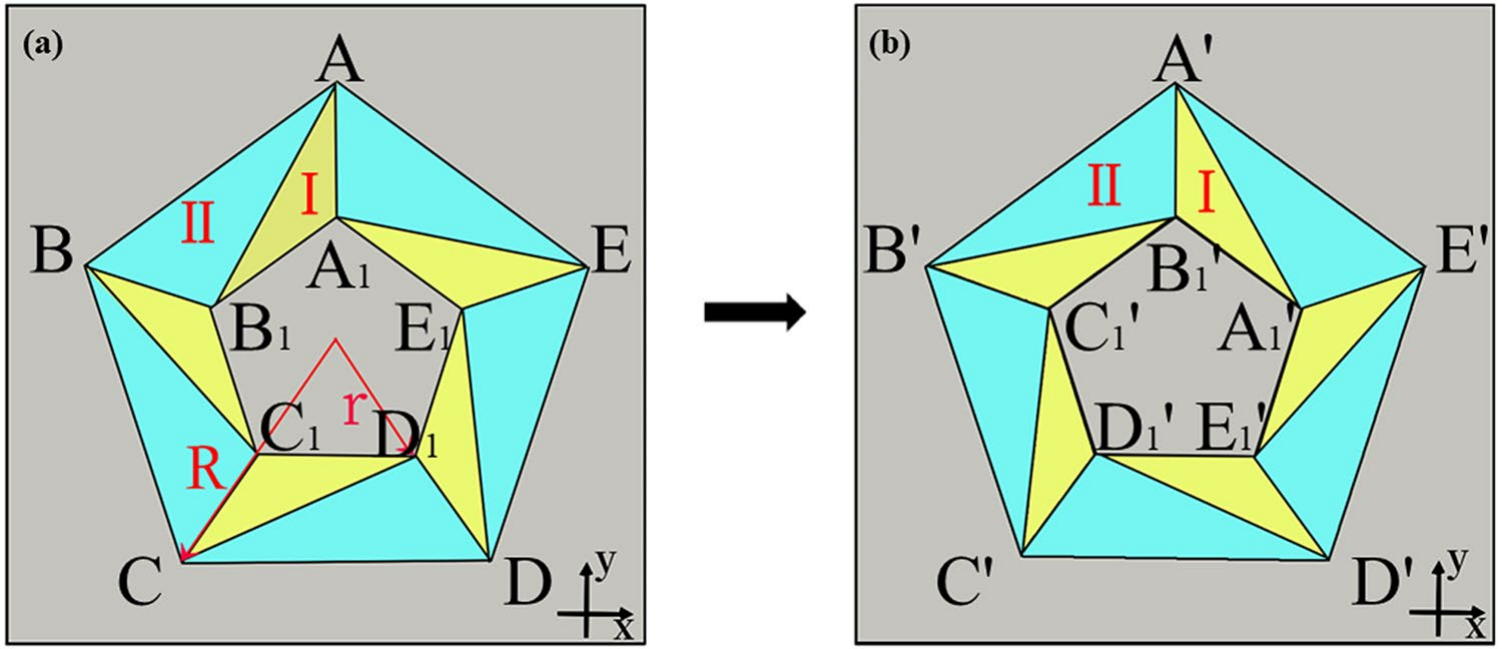
Schematic of coordinate transformation in the design of acoustic rotator. (**a**) Virtual space, R and r mean the radii of outer and inner pentagons respectively. (**b**) Physical space.

Next, we apply the linear transformation method to the second space transformation mode without stretching or compression, and design the acoustic rotator to explore whether the parameters are still homogeneous. In Fig. 3, we schematically illustrate the design concept of an N-sided regular polygonal rotator. Figure 3a means the virtual space before transformation and Fig. 3b represents the physical space. For the whole device, it is that the internal small pentagon rotates $2\pi /n = 72^\circ$ around its center. *n* represents the number of sides of a regular polygon rotator. Then, the propagation path inside central area would rotate at the same angle. For the case of pentagonal acoustic rotator, the transformation denoted that the propagations of acoustics rotate at the quintile of centroid angle while outer space stayed unchanged.

Rotator’s transformation equations are different from concentrator. Equations are set by undetermined coefficient method showing the coordinate relationship between virtual space and physical space for region I and II.

5 $$ \begin{aligned} & u_{i}^{{\prime}} = A_{1} u_{i} + B_{1} v_{i} + C_{1} \\ & v_{i}^{{\prime}} = A_{2} u_{i} + B_{2} v_{i} + C_{2} \\ & z^{\prime} = z \\ \end{aligned} $$

$A_{1} ,B_{1} ,C_{1} ,A_{2} ,B_{2} ,C_{2}$ are coefficients of equations. The detailed discussion on the transformation can be found in Supplementary Materials.

## Results

To demonstrate the device effect, we exemplified the concentrators and rotators. A finite element method based solver COMSOL Multiphysics is used to validate the performance of designed concentrators and rotators. First, we carried out the simulations of regular quadrilateral concentrator. Here, the geometric parameters were selected as $L = 0.6\;{\text{m}},{ }L_{0} = 0.4\;{\text{m}}$ and $L_{1} = 0.2\;{\text{m}}$ in Fig. 1. The background medium is water, and its mass density and bulk modulus are $\rho_{0} = 1000\;{\text{kg/m}}^{3}$, ${\text{B}}_{0} = 2.5 \times 10^{9} \;{\text{Pa}}$ while the frequency is 7.5 kHz.

**Figure 4:**
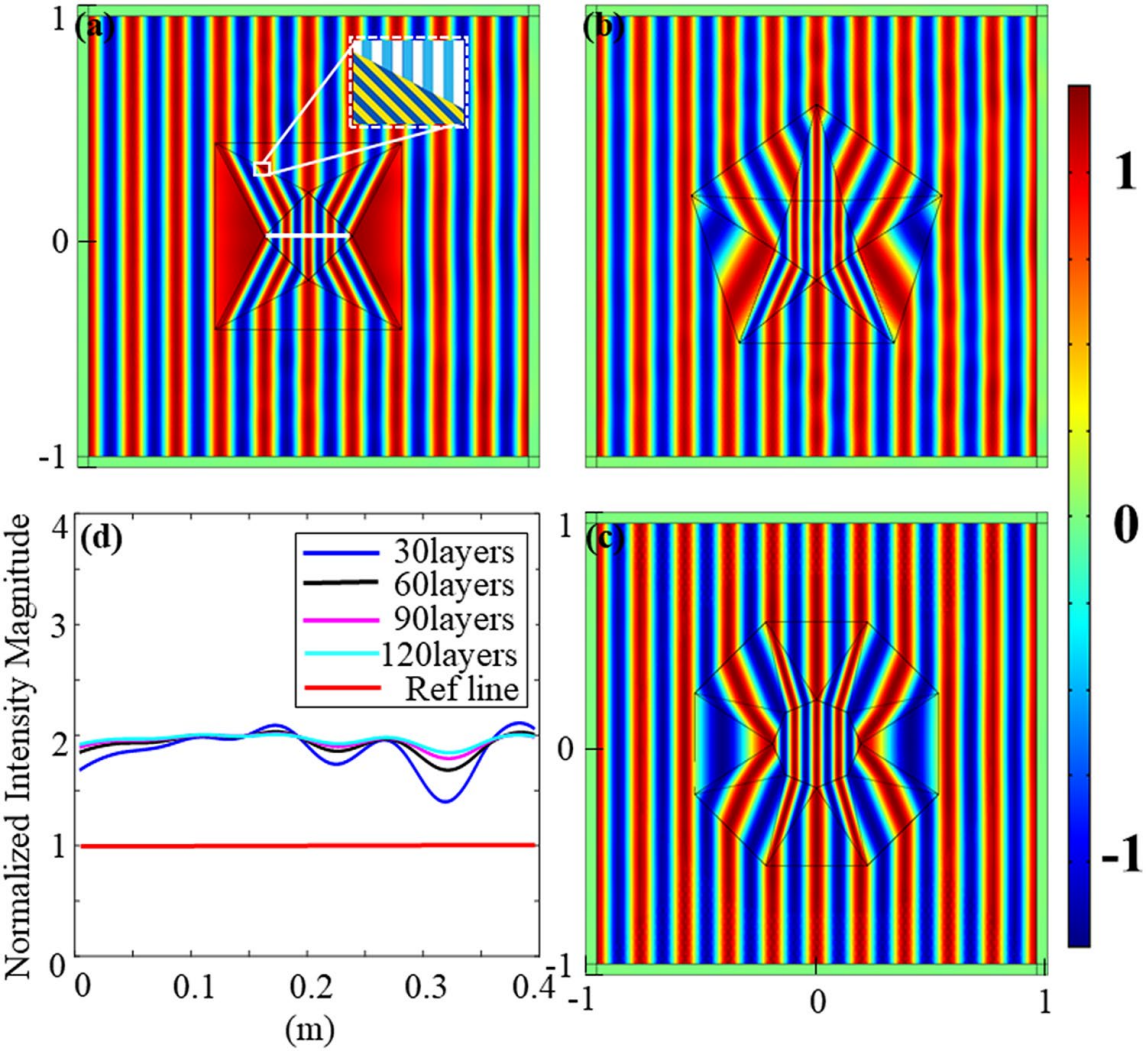
(**a**–**c**) Pressure field distributions of different regular polygons. (**a**) Square concentrator; (**b**) pentagonal concentrator; (**c**) octagonal concentrator; (**d**) normalized intensity magnitude in central area of (**a**). The values are taken from the straight line segments of ${\text{y}} = 0\;{\text{m}}$ and ${\text{x}} = - 0.2\sim 0.2\;{\text{m}}$. The line is located in the central area, as shown by the white line segment in (**a**). The Ref line is reference line in the same position, but without the device. Curves in different colors represent the pressure fields of different layers.

For quadrilateral concentrator, the constitutive parameters were calculated using aforesaid methods. From Eqs. (1) and (2), we can find that as long as $L$, $L_{0}$ and ${\text{L}}_{1}$ are determined, $k_{1}$, $k_{2}$ and $k_{3}$ are constant values accordingly. Therefore, $\rho_{u}$, $\rho_{v}$, ${\text{B}}_{1}$ and ${\text{B}}_{2}$ are constant, so are $\rho_{1}$ and $\rho_{2}$. The parameters of two kinds of subdomains are simplified into two sets, so four materials can form segment I and segment II. In geometric model, segment I and segment II were both split into many units where each unit consisted of two alternating material at different densities. Figure 4a–c show the pressure fields distribution under acoustic plane wave irradiation in different geometric shapes. It was observed that the acoustic wave in the designed device was concentrated by the concentrator into the compressive region i.e., the internal square.

Take the case of square concentrator as an example. Based on these geometric parameters, mass densities and bulk modulus are calculated as:

6 $$ \begin{aligned} & {\text{For}}\;{\text{ region}}\;{\text{ I}}:\quad \quad \rho_{1}^{{{\text{I}}^{\prime }}} = 5.61\rho_{0} ,\quad \rho_{2}^{{{\text{I}}^{\prime }}} = 0.18\rho_{0} ,\quad {\text{B}}^{{{\text{I}}^{\prime }}} = 0.72{\text{B}}_{0} \\ & {\text{For}}\;{\text{ region}}\;\;{\text{ II:}}\quad \quad \rho_{1}^{{{\text{II}}^{\prime }}} = 18.43\rho_{0} ,\quad \rho_{2}^{{{\text{II}}^{\prime }}} = 0.05\rho_{0} ,\quad {\text{B}}^{{{\text{II}}^{\prime }}} = 9.24{\text{B}}_{0} \\ & {\text{For }}\;{\text{central}}\;{\text{ square:}}\quad \quad \rho_{1}^{{{\text{c}}^{\prime }}} = \rho_{0} ,\quad \rho_{2}^{{c{\prime }}} = \rho_{0} ,\quad {\text{B}}^{{c^{\prime }}} = 0.25{\text{B}}_{0} \\ \end{aligned} $$

According to the aforementioned approach, each region consisted of two kinds of material with the same bulk modulus but different densities. Layered structures are arranged following their local u direction while the solid frame was hidden in Comsol, as shown in Figs. 2c and 4a. Other regular polygonal concentrators’ simulations are obtained by repeating the same process and shown in Fig. 4b,c.

Figure 4 shows the results and performance of square, regular pentagon and octagon concentrators. We can observe that the incident waves in central area are compressed without any scattering. Thus, the energy intensity of the central part should be greater than that of the peripheral part. Figure 4d is the normalized intensity in central area of Fig. 4a. As the number of layers increases, the fluctuation of the middle part of the curve becomes smaller, that is, the energy intensity becomes more stable. As a resultant, a multilayer physical realization approach with homogeneous materials gives a good performance. Unlike inhomogeneous materials, we just simplify the parameters and transform them into a few feasible materials.

We also conducted numerical simulations of acoustic rotator in COMSOL Multiphysics. Same as concentrators’ simulation, the background medium is water, and its mass density and bulk modulus are $\rho_{0} = 1000\;{\text{kg/m}}^{3}$, ${\text{B}}_{0} = 2.5 \times 10^{9} \;{\text{Pa}}$ while the frequency was 7.5 kHz, $R = 0.6\;{\text{m}}$ and $r = 0.3\;{\text{m}}$ in Fig. 3. The layered structure of the rotator is still arranged along the u direction of each segment’s local coordinate system. But the difference is that the density tensors of the rotator has coupling components in the u and v directions. A rotation of the local system is necessary to realize the diagonalization of the density tensor. And each segment’s layered structure is arranged along the direction of the new coordinate system after diagonalization. Then, simulations make the effects more visualized. Inside the device, incident beams are bent at a certain angle without perturbing the exterior fields.

Physical realization can be achieved by applying effective medium theory. Furthermore, each segment’s constitutive parameters in Fig. 3 are expressed as:

7 $$ \begin{aligned} & {\text{For}}\;{\text{
region}}\;{\text{I:}}\quad \quad \rho_{1}^{{{\text{I}}^{\prime }}} =
7.57\rho_{0} ,\quad \rho_{2}^{{{\text{I}}^{\prime }}} = 0.13\rho_{0}
,\quad {\text{B}}^{{{\text{I}}^{\prime }}} = {\text{B}}_{0} \\ &
{\text{For }}\;{\text{region }}\;{\text{II:}}\quad \quad
\rho_{1}^{{{\text{II}}^{\prime} }} = 20.65\rho_{0} ,\quad
\rho_{2}^{{{\text{II}}^{\prime }}} = 0.05\rho_{0} ,\quad
{\text{B}}^{{{\text{II}}^{\prime }}} = {\text{B}}_{0} \\ &
{\text{For}}\;{\text{ centralarea:}}\quad \quad
\rho_{1}^{{{\text{c}}^{\prime }}} = \rho_{0} ,\quad
\rho_{2}^{{c^{\prime }}} = \rho_{0} ,\quad {\text{B}}^{{c^{\prime
}}} = {\text{B}}_{0} \\ \end{aligned} $$

**Figure 5:**
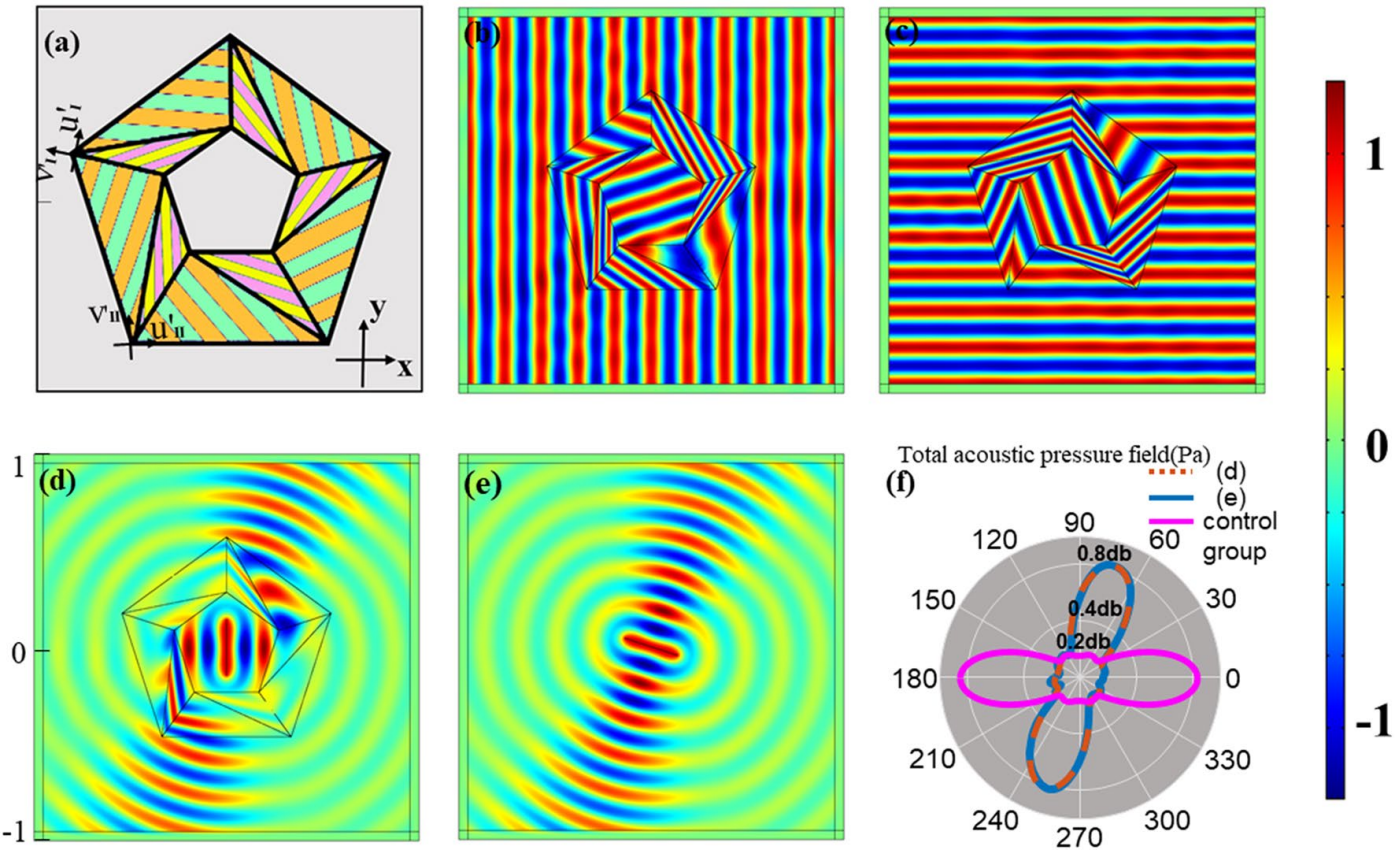
(**a**) The layered structure schematic of acoustic rotator; (**b**) horizontal acoustic pressure field distribution at 60 layers; (**c**) vertical acoustic pressure field distribution at 120 layers; (**d**) acoustic pressure field distribution at the internal line source. The emission direction of the source is horizontal while the antenna is vertical, but the external waves rotate 72 degrees counterclockwise; (**e**) acoustic pressure field distribution at the internal line source without transformation. The source rotates anticlockwise at an angle $\upalpha  = 72^\circ { }$ relative to the angle of (**d**). The emission direction is 72 degrees while the antenna is perpendicular to it; (**f**) total acoustic pressure field at a distance of 0.6 m from the center. Numerical detections collect (**d**, **e**) and the case whose antenna is vertical without coordinate transformation.

Figure 5 shows the layered structure effects and simulation results of regular pentagonal rotators. Figure 5a denotes the geometry structure of rotator and orientation of layered structure. In Fig. 5b,c, acoustic waves are incident on device from vertical and horizontal directions respectively. When waves radiate into device, they deflect at different angles in different regions. The number of layers also affect the rotator’s performance. We can observe that Fig. 5b has a slight disturbance while Fig. 5c does not since the layers of Fig. 5b is 60 while Fig. 5c is 120. Similar to concentrator, layered structure simplifies the parameters of this rotator and makes the fabrication achievable. More layered structure would cause better effect.

In addition, its results are also verified when source is inside. Figure 5d–f show the effects of rotator visually by changing the angle of the antenna and detecting the numerical diagram of the total acoustic pressure field. From Fig. 5d, the acoustic waves would be deflected within the rotator. Then, the direction outside the device is 72 degrees from the original direction. In another case, as shown in Fig. 5e, when the internal antenna rotates 72 degrees anticlockwise, the effect of the device will be offset so that the waves still enter the medium the same as Fig. 5d. At 0.6 m away from the center, we detected the value of the total acoustic pressure fields of Fig. 5d,e. Besides, we conducted a control group as a comparison. The control group was similar to the Fig. 5d, but there was no rotator here. We observe that the detection data of Fig. 5d,e are almost identical while case of control group is just horizontal. In other words, processed by rotator, the external pressure field distribution of Fig. 5d is the same as the natural case of Fig. 5e. At the same time, it can be concluded from the comparison that, the device can rotate the propagation direction regardless of the source's position. Therefore, the rotator, whether as a transmitter or a receiver, has good rotation effects.

## Discussion

In summary, we have proposed the design of acoustic concentrators and rotators with linear coordinate transformation. The coordinate transformation method realizes the homogenization and diagonalization of the constitutive parameters. Homogeneous parameters are easier to achieve. It should be noted that, although the requested parameters are still unachievable for practical realization, the difficulties of its implementation is great simplified. Furthermore, the excellent characteristics and effectiveness are confirmed via numerical simulations with layered structures. In acoustic concentrator and acoustic rotator, only five kinds of materials with homogeneous parameters are needed to realize the fabrication of devices. The device realization becomes feasible with few homogenous materials.

## Supplementary Information

Supplementary Information.

## References

[1] Pendry JB, Schurig D, Smith DR (2006). Controlling electromagnetic fields. Science.

[2] McCall M (2018). Roadmap on transformation optics. J. Opt..

[3] Leonhardt U (2006). Optical conformal mapping. Science.

[4] Schurig D (2006). Metamaterial electromagnetic cloak at microwave frequencies. Science.

[5] Sun F, Zhang YJ, Evans JL, He SL (2019). A camouflage device without metamaterials. Prog. Electromagn. Res..

[6] Xi S, Chen HS, Wu BI, Kong JA (2009). One-directional perfect cloak created with homogeneous materials. IEEE Microw. Wirel. Compon. Lett..

[7] Lai Y (2009). Illusion optics: the optical transformation of an object into another object. Phys. Rev. Lett..

[8] Madni HA (2017). Non-contact method to freely control the radiation patterns of antenna with multi-folded transformation optics. Sci. Rep..

[9] Sun F (2017). Transformation optics: from classic theory and applications to its new branches. Laser Photon. Rev..

[10] Wang HP (2017). Panoramic lens designed with transformation optics. Sci. Rep..

[11] Jiang ZH, Gregory MD, Werner DH (2011). Experimental demonstration of a broadband transformation optics lens for highly directive multibeam emission. Phys. Rev. B.

[12] Chen Y (2014). Two-dimensional concentrators using transformation optics via rotated-layered systems. Microw. Opt. Technol. Lett..

[13] Yang CF, Huang M, Yang JJ, Mao FC (2018). Homogeneous multifunction devices designing and layered implementing based on rotary medium. Opt. Express.

[14] Cummer SA, Christensen J, Alù A (2016). Controlling sound with acoustic metamaterials. Nat. Rev. Mater..

[15] Milton G, Briane M, Willis J (2006). On cloaking for elasticity and physical equations with a transformation invariant form. N. J. Phys..

[16] Cummer S, Schurig D (2007). One path to acoustic cloaking. N. J. Phys..

[17] Chen H, Chan C (2010). Acoustic cloaking and transformation acoustics. J. Phys. D Appl. Phys..

[18] Rahm M (2008). Design of electromagnetic cloaks and concentrators using form-invariant coordinate transformations of Maxwell’s equations. Photonics Nanostruct. Fundam. Appl..

[19] Chen H, Chan C (2014). Transformation media that rotate electromagnetic fields. Appl. Phys. Lett..

[20] Yang J, Huang M, Yang C, Cai G (2011). A metamaterial acoustic concentrator with regular polygonal cross section. J. Vib. Acoust. Trans. ASME.

[21] Jiang X, Liang B, Zou X, Yin L, Cheng J (2014). Broadband field rotator based on acoustic metamaterials. Appl. Phys. Lett..

[22] Li TH, Huang M, Yang JJ, Li YL, Yu J (2012). Diamond-shaped acoustic concentrator with homogeneous material parameters. Acoust. Phys..

[23] Wang YR, Zhang H, Zhang SY, Fan L, Sun HX (2014). Broadband acoustic concentrator with multilayered alternative homogeneous materials. J. Acoust. Soc. Am..

[24] Yang JJ, Huang M, Yang CF, Cai GH (2011). A metamaterial acoustic concentrator with regular polygonal cross section. J. Vib. Acoust. Trans. ASME.

[25] Lu WJ, Jia H, Bi YF, Yang YZ, Yang J (2017). Design and demonstration of an acoustic right-angle bend. J. Acoust. Soc. Am..

[26] Wood B, Pendry JB, Tsai DP (2006). Directed sub-wavelength imaging using metal-dielectric system. Phys. Rev. B.

