# Supplementary material for: Homogeneous material based acoustic concentrators and rotators with linear coordinate transformation
Source: Sci Rep. 2021 Jun 1;11:11531. doi: 10.1038/s41598-021-91146-x (PMC8169701; doi:10.1038/s41598-021-91146-x)
Supplement: Supplementary file 1 — Supplementary Information. [file 41598_2021_91146_MOESM1_ESM.docx]

Supplementary Material for

Homogeneous Material based Acoustic Concentrators and Rotators with Linear Coordinate Transformation

**Huaping Wang**^1*^**,** **Lei Zhang**^1^**, Shahnawaz Shah**^2^**, Rongrong Zhu**^3^**, and Bin Zheng**^2*^

^1^ Key Laboratory of Ocean Observation-Imaging Testbed of Zhejiang Province, Institute of Marin Electronics Engineering, Ocean College Zhejiang University, Hangzhou, 310058, P. R. China

^2^ College of Information Science & Electronic Engineering Zhejiang University, Hangzhou, 310058, P. R. China

^3^ School of Information & Electrical Engineering, Zhejiang University City College, Hangzhou, 310015, P. R. China

*corresponding. B. Z. (email: [zhengbin@zju.edu.cn)](mailto:zhengbin@zju.edu.cn)) and H. W. (email: [hpwang@zju.edu.cn)](mailto:hpwang@zju.edu.cn))

**I. Transformation for three dimensional acoustic concentrator**

In the following, we discuss the 3D case of concentrator for example. The shape of the device is regular hexahedron, and the central region is regular octahedron. By connecting the vertices of hexahedron and octahedron, we divide the device into 26 sub- regions and a central region. Similar to the 2D case, sub regions can be divided into three categories: Region I is a pyramid, which share the bottom surface with the hexahedron concentrator’s surface, and share the vertices with the central octahedron region’s vertices. Region II is a tetrahedron that composed of vertices of regular hexahedron and surfaces of regular octahedron, as shown in the blue area of Fig. S1(a). And region III is composed of edges of regular hexahedron and octahedron, as shown in the red area of Fig. S1(a). The octahedron in the central region is the basis and purpose of all transformations. It is spatially compressed from Fig. S1(a) to Fig. S1(b). Fig. S1(a) shows the virtual space of concentrator and Fig. S1(b) shows the physical space. After transformation of large octahedron in virtual space to small octahedron in physical space, the internal space of octahedron can be concentrated and focused. We can extend the transformation equation to hexahedral concentrator.

For region I: $u_{a}^{'}=u_{a}, v_{a}^{'}=v_{a}, w_{a}^{'}=k_{a}w_{a}$

For region II: $u_{b}^{'}={k_{b}u}_{b}, v_{b}^{'}={k_{b}v}_{b}, w_{b}^{'}={k_{c}w}_{b}$ S1

For region III: $u_{c}^{'}=u_{c}, v_{c}^{'}={k_{b}v}_{c}, w_{c}^{'}={k_{d}w}_{c}$

For central region: $u_{d}^{'}=u_{d}/k_{b}, v_{c}^{'}=v_{d}/k_{b}, w_{c}^{'}=w_{d}/k_{b}$

where $k_{a}$, $k_{b}$ and $k_{c}$ are compression ratios of corresponding regions: $k_{a}=\frac{\left( \frac{1}{2} \right)L_{1}-\left( \frac{\sqrt{2}}{2} \right)L_{3}}{\left( \frac{1}{2} \right)L_{1}-\left( \frac{\sqrt{2}}{2} \right)L_{2}}, k_{b}=\frac{L_{3}}{L_{2}}, k_{c}=\frac{\left( \frac{\sqrt{3}}{2} \right)L_{1}-\left( \frac{\sqrt{6}}{6} \right)L_{3}}{\left( \frac{\sqrt{3}}{2} \right)L_{1}-\left( \frac{\sqrt{6}}{6} \right)L_{2}},k_{d}=\frac{\left( \frac{\sqrt{2}}{2} \right)L_{1}-\left( \frac{1}{2} \right)L_{3}}{\left( \frac{\sqrt{2}}{2} \right)L_{1}-\left( \frac{1}{2} \right)L_{2}}.$ $L_{1}$ and $L_{2}$ denote the edge length of regular hexahedron and octahedron in virtual space respectively, while $L_{3}$ is the edge length of regular octahedron in physical space, as shown in Fig. S1.


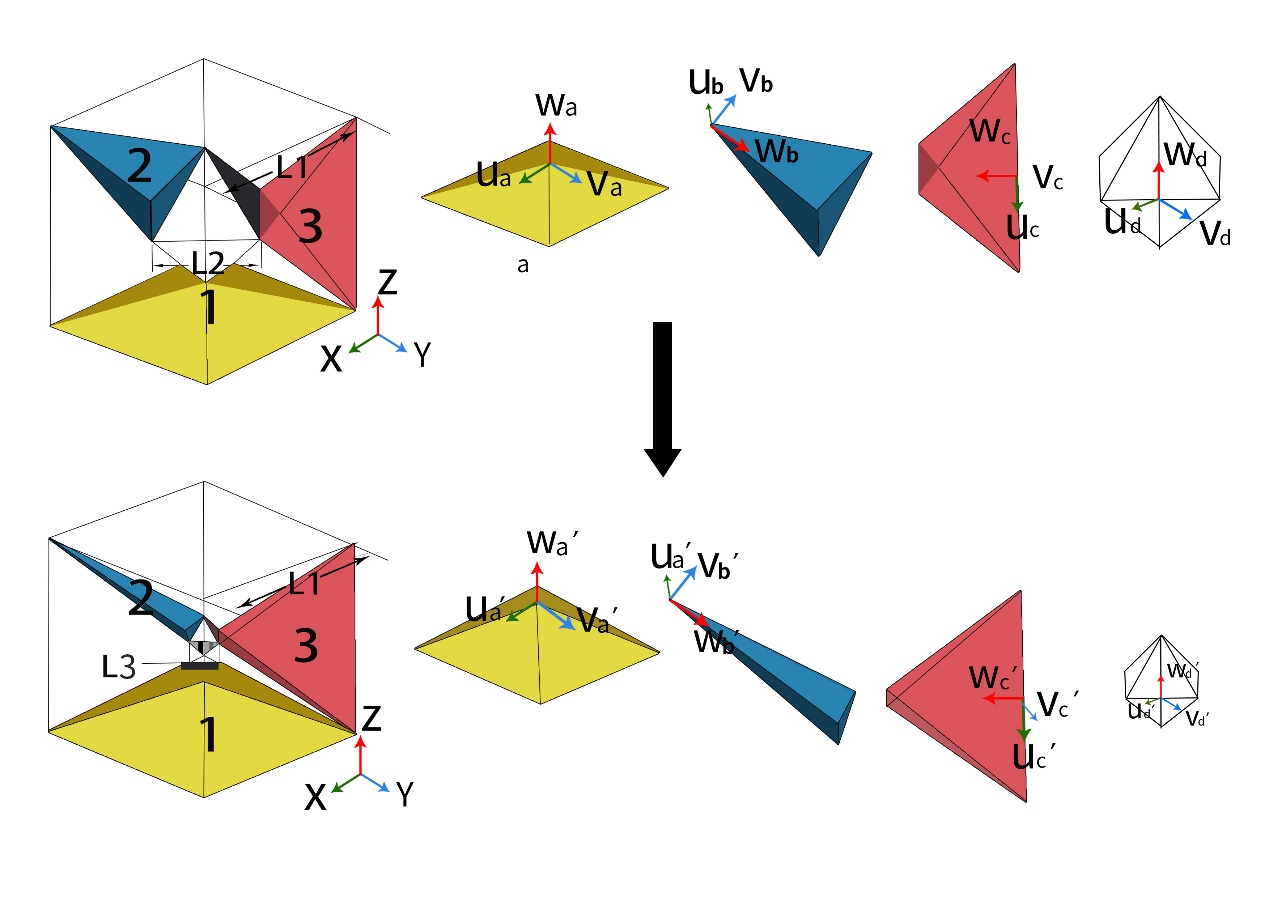


**Figure S1.** (a) Virtual space of 3D acoustic concentrator; (b) Physical space of 3D acoustic concentrator.

According to the transformation functions of Eq. S1, the density tensor $\rho$ and bulk modulus B of each sub-region can be calculated as,

For region I: $\rho_{u}^{I^{'}}=\rho_{0}k_{a}, \rho_{v}^{I^{'}}=\rho_{0}k_{a}, \rho_{w}^{I^{'}}=\frac{\rho_{0}}{k_{a}}, B^{I^{'}}=k_{a}B_{0}$

For region II: $\rho_{u}^{\mathrm{II}^{'}}=\rho_{0}k_{c}, \rho_{v}^{\mathrm{II}^{'}}=\rho_{0}k_{c}, \rho_{w}^{\mathrm{II}^{'}}=\rho_{0}\frac{k_{b}}{k_{c}}, B^{\mathrm{II}^{'}}=B_{0}{k_{b}^{2}k}_{c}$ S2

For region III: $\rho_{u}^{\mathrm{III}^{'}}=k_{b}k_{d}\rho_{0}, \rho_{v}^{\mathrm{III}^{'}}=k_{d}\rho_{0}, \rho_{v}^{\mathrm{III}^{'}}=k_{b}\rho_{0}, B^{\mathrm{III}^{'}}=B_{0}k_{b}k_{d}$

For central region: $\rho_{u}^{c^{'}}=\rho_{0}/k_{b}, \rho_{v}^{c^{'}}=\rho_{0}/k_{b}, \rho_{v}^{c^{'}}=\rho_{0}/k_{b}, B^{c^{'}}=B_{0}/k_{b}^{3}$

Obviously, three-dimensional concentrator only need four kinds of homogeneous anisotropic media to be constructed. Compared with inhomogeneous case, the difficulty of constructing 3D concentrator is greatly reduced. They can also be constructed by layered structure. Therefore, concentrator and rotator based on homogeneous coordinate transformation have more simplified parameters and are easier to construct in practical applications.

**II. Transformation for acoustic rotator**


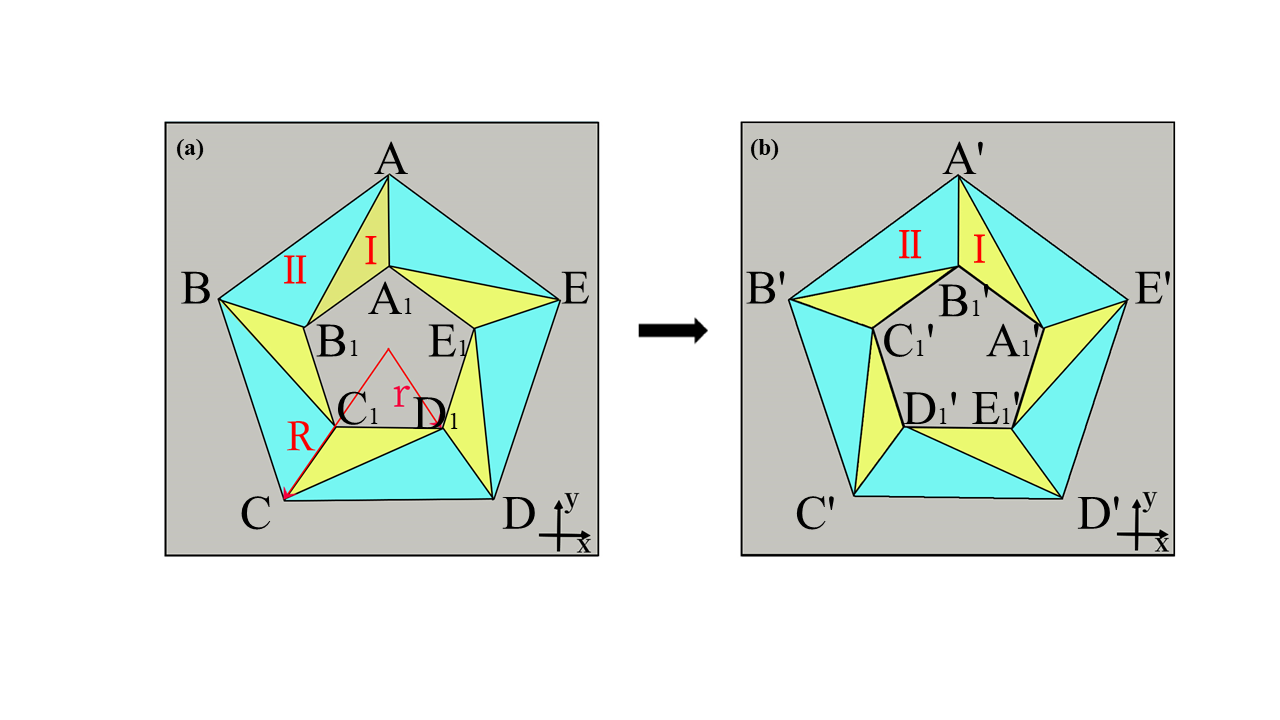


**Figure S2.** Schematic of coordinate transformation in the design of acoustic rotator. (a) Virtual space, R and r mean the radii of outer and inner pentagons respectively. (b) Physical space.

In the following, we explain the transformation process of each region. In Fig. S2, we schematically illustrate the design concept of an N-sided regular polygonal rotator. Similarly, Fig. S2(a) means the virtual space before transformation and S2(b) represents the physical space. First, the internal area was split into annular region and central region to set stage for coordinate transformation. Specifically, taking a group of annular region as an example, the $\Delta AA_{1}B_{1}$ and $\Delta ABB_{1}$ of virtual space in Fig. S2(a) were mapped into $\Delta A{'A}_{1}'B_{1}'$ and $\Delta A'B{'B}_{1}'$ of physical space in Fig. S2(b) respectively. For $\Delta AA_{1}B_{1}$ of Fig. S2(a) and $\Delta A'A_{1}'B_{1}'$ of Fig. S2(b), the mapping relationship was: $A_{1}$ and $B_{1}$ of Fig. S2(a) to $A_{1}'$ and $B_{1}'$ of Fig. S2 (b). In the same way, $AA_{1}E_{1}E$, $EE_{1}D_{1}D$, $DD_{1}C_{1}C$, and $CC_{1}B_{1}B$ of virtual space in Fig. S2(a) were mapped into $A{'A}_{1}'E_{1}'E'$, $E'E_{1}'D_{1}'D'$, $D'D_{1}'C_{1}'C'$, and $C'C_{1}'B_{1}'B'$ of physical space in Fig. S2(b) respectively. Therefore, the internal small pentagon rotates $2\pi/n$ around its center. *n* represents the number of sides of a regular polygon rotator. Then, the propagation path inside central area would rotate at the same angle. For the case of pentagonal acoustic rotator, the transformation denoted that the propagations of acoustics rotate at the quintile of centroid angle while outer space stayed unchanged.

The transformation method of acoustic rotator is linear transformation. The transformation equations of region I and II are shown in the following:

$u_{i}^{'}=A_{1}u_{i}+B_{1}v_{i}+C_{1}$

$v_{i}^{'}=A_{2}u_{i}+B_{2}v_{i}+C_{2}$ S3

$z'=z$

$A_{1}{,B}_{1},C_{1},A_{2}{,B}_{2},C_{2}$ are coefficients of equations. The vertex coordinates of each small triangle in Fig. 3 are substituted into Eq. S(3). Then, the value of the coefficient can be calculated. Taking $AA_{1}BB_{1}$ as an example, when $R=0.6m$ and $r=0.3m$, the coordinates of the three vertices of $\Delta AA_{1}B_{1}$ and $\Delta A{'A}_{1}^{'}B_{1}'$ are: $A\left( 0,0.6 \right), A_{1}\left( 0,0.3 \right), B_{1}\left( -0.29,0.09 \right), B\left( -0.58,0.18 \right), A{'\left( 0,0.6 \right), A}_{1}^{'}\left( 0.29,0.09 \right), B_{1}^{'}\left( 0,0.3 \right), B^{'}\left( -0.58,0.18 \right).$ Then, the coefficients $A_{1}{,B}_{1},C_{1},A_{2}{,B}_{2},C_{2}$ of $\Delta AA_{1}B_{1}$ and $\Delta A{'A}_{1}'B_{1}'$ can be obtained: $A_{1}=0.309, B_{1}=-0.951, C_{1}=0.571, A_{2}=0.502, B_{2}=1.691, C_{2}=-0.415$. In the same way, the coefficients of $\Delta ABB_{1}$ and $\Delta A'B{'B}_{1}'$ can be calculated: $A_{1}=0.837, B_{1}=1.678, C_{1}=-0.218, A_{2}=0.674, B_{2}=2.545, C_{2}=-0.671$. We get the Jacobian matrix $J$ of different regions, which is used to calculate the densities and bulk modulus. Then, densities and bulk modulus can be calculated by following equation:

${\rho'}^{-1}=\frac{J\rho_{0}^{-1}J^{T}}{\left| J \right|}$

$B'=\left| J \right|B_{0}$ S4

The density tensor and bulk modulus can be obtained. However, the elements outside the diagonal of the density tensor are nonzero. We use the rotating coordinate system to diagonalize it. The density component after diagonalization and bulk modulus are: $\rho_{I\_u}=259.62kg/m^{3},$ $\rho_{I\_v}=3851.8kg/m^{3}$, $B_{I}=2.25\times{10}^{9}Pa$, $\rho_{\mathrm{II}\_u}=96.626kg/m^{3}$, $\rho_{\mathrm{II}\_v}=10349kg/m^{3}$, $B_{\mathrm{II}}=2.25\times{10}^{9}Pa$. Next, parameters need to be replaced by the density and bulk modulus of the layered structure. According to Eqs. (3) and (4), the densities and bulk modulus for each layered structure can be obtained. Likely, by using the effective medium theory, we can simplify the parameters to obtain. When $\rho_{0}=1000kg/m^{3}$, $B_{0}=2.5\times{10}^{9}Pa$, the densities and bulk modulus are as follows:

For region I: ${\rho_{1}^{I}}^{'}=7.571.6kg/m^{3},{\rho_{2}^{I}}^{'}=132.07kg/m^{3},{B^{I}}^{'}=2.5\times{10}^{9}Pa$

For region II: ${\rho_{1}^{\mathrm{II}}}^{'}=20650kg/m^{3},{\rho_{2}^{\mathrm{II}}}^{'}=48.426kg/m^{3},{B^{\mathrm{II}}}^{'}=2.5\times{10}^{9}Pa$ S5

For central area: ${\rho_{1}^{c}}^{'}=\rho_{0},{\rho_{2}^{c}}^{'}=\rho_{0},{B^{c}}^{'}=2.5\times{10}^{9}Pa$
